# Earlier chronotype in patients with rheumatoid arthritis

**DOI:** 10.1007/s10067-020-05546-x

**Published:** 2021-01-16

**Authors:** G. Esther A. Habers, Annette H. M. van der Helm-van Mil, Dieuwke S. Veldhuijzen, Cornelia F. Allaart, Erno Vreugdenhil, Daniëlle E. J. Starreveld, Tom W. J. Huizinga, Andrea W. M. Evers

**Affiliations:** 1 Health, Medical, and Neuropsychology Unit, Institute of Psychology, Leiden University, Wassenaarseweg 52, 2333 Leiden, AK the Netherlands; 2 Leiden Institute for Brain and Cognition, Leiden, the Netherlands; 3 Department of Rheumatology, Leiden University Medical Center, Leiden, the Netherlands; 4 Department of Cell and Chemical Biology/Department of Dermatology, Leiden University Medical Center, Leiden, the Netherlands; 5 Department of Psychosocial Research and Epidemiology, Netherlands Cancer Institute, Amsterdam, the Netherlands; 6 Department of Psychiatry, Leiden University Medical Center, Leiden, the Netherlands; 7 Industrial Design Engineering, Delft University of Technology, Delft, the Netherlands; 8 Erasmus School of Health Policy and Management, Erasmus University, Rotterdam, the Netherlands

**Keywords:** Chronobiology, Chronotype, Disease activity, Patient-reported outcomes, Rheumatoid arthritis

## Abstract

**Objectives:**

Rheumatoid arthritis (RA) patients show an earlier circadian rhythm (i.e. serum melatonin peaks earlier during the night, indicating an earlier timing of the internal circadian pacemaker). In the current study, we examined whether the chronotype, which is influenced by the circadian rhythm, is also earlier. In addition, we explored whether chronotype is related to disease activity and patient-reported outcomes.

**Methods:**

The chronotype (Munich Chronotype Questionnaire) of patients with RA (*n* = 121; mean age 60 years, 73% female) was compared with that of subjects from the general population (norm group; *n* = 1695) with a one-sample *t* test. In addition, we investigated chronotype in relation to disease activity (Disease Activity Score; DAS), reported morning stiffness, fatigue (Checklist Individual Strength), and health-related quality of life (RAND-36).

**Results:**

The chronotype of patients with RA was, on average, 23 min (95% CI, 15 to 31 min) earlier than that of the norm group (*t*(115) = − 5.901, *p* < 0.001, *d* = 0.55). Chronotype was not related to disease activity or patient-reported outcomes (*p* > 0.05).

**Conclusion:**

As expected, chronotype was earlier in RA patients. However, in this correlational study, chronotype was not related to disease activity or patient-reported outcomes. An experimental study is needed to examine whether delaying the circadian rhythm has a positive influence on these outcomes. This insight could improve our understanding of the pathophysiology of RA and contribute to exploring new treatment possibilities.

**Table:** 

Key Points *• This is the first study examining chronotype in patients with rheumatoid arthritis, and how chronotype relates to disease activity and patient-reported outcomes.* *• We found an earlier chronotype in patients with rheumatoid arthritis than in subjects from the general population.* *• In this correlational study, chronotype was not related to disease activity or patient-reported outcomes. An experimental study is needed to examine whether delaying the circadian rhythm positively influences these outcomes.*

**Supplementary Information:**

The online version contains supplementary material available at 10.1007/s10067-020-05546-x.

## Introduction

Chronobiological research in relation to rheumatoid arthritis (RA) goes back several decades [1] and continues to be performed to improve our understanding of the pathophysiology of RA. It has been noted, for instance, that shift work is associated with an increased risk for developing RA [2]. Furthermore, daily fluctuations have been observed in patients’ symptoms (e.g. pain and stiffness), with most pain and stiffness experienced in the early morning. The underlying cause of this morning peak in symptom severity seems to be the increase of pro-inflammatory cytokines during the night. One finding that supports this idea is that administration of glucocorticoids during the night is indeed more effective than morning treatment [3].

Circadian rhythms are endogenous rhythms that follow approximately a 24-h cycle. The rhythms are entrained to the environment by zeitgebers, such as the light-dark cycle. Well-functioning circadian rhythms are essential for the healthy functioning of all bodily systems. An important signalling hormone of the circadian rhythm is melatonin. It is directed by the suprachiasmatic nucleus, which is the internal central circadian pacemaker.

Circadian rhythm alterations are thought to play an important role in the pathophysiology of RA [3]. It has been shown that in patients with RA serum melatonin peaks earlier in the night [4], indicating an earlier timing of the circadian rhythm. Furthermore, circadian rhythms of some immune cell populations were found to be absent, new [5], or enhanced [6] in patients with active RA, compared to those in healthy subjects.

Circadian rhythms are one of the factors that influence an individual’s chronotype. Each individual’s chronotype reflects the phase of entrainment, which is the relation between the internal day and external day. The chronotype can be calculated from sleep times: often the half-way point between sleep onset and sleep end (i.e. mid-sleep) is used as a measure of a subject’s chronotype. It has not yet been explored whether the chronotype is different in patients with RA than in subjects from the general population. Insight into the chronotype and its relation with disease activity and patient-reported outcomes can contribute to our understanding of the pathophysiology of RA. This provides a possible starting point for exploring new treatment possibilities.

Therefore, in the current study, the first aim was to examine whether the chronotype was earlier in patients with RA than in subjects from the general population. Given the strong relation between the timing of melatonin onset and mid-sleep in the general population [7], and given that melatonin peaks were earlier in patients with RA [4], we expected to find an earlier chronotype in patients with RA than in subjects from the general population.

Secondly, we explored whether chronotype was related to disease activity and to patient-reported outcomes (i.e. reported morning stiffness, fatigue, and health-related quality of life (HR-QoL)). Thirdly, for explorative reasons, we also assessed the presence in the RA population of self-assessed sleep disorders, including circadian rhythm sleep disorders.

## Materials and methods

### Study design and subjects

The study had a cross-sectional correlational design. The population-based prospective Leiden Early Arthritis Clinic (EAC), set up in 1993 [8], includes patients with confirmed arthritis (after physical examination) and a symptom duration of less than 2 years at the moment of inclusion in the EAC. The inclusion and exclusion criteria for this cohort have been published previously [8]. For this sub-study, additional exclusion criteria included being unable to give informed consent and/or lacking sufficient understanding of Dutch. Patients received an email or, if no email address was available, a postal letter with information about the current study and an informed consent page/form. The Medical Ethical Committee of the Leiden University Medical Center waived ethical approval; the study was carried out in compliance with the Helsinki Declaration. Data were collected in the period 8–24 October 2015 (daylight saving time).

### Measurements

#### Questionnaires for primary data collection

The questionnaires filled in by the subjects included the Munich Chronotype Questionnaire, the Checklist Individual Strength-20, RAND-36, and the Holland Sleep Disorders Questionnaire. We also asked questions about the educational level, marital status, and medication use.

##### Munich Chronotype Questionnaire (MCTQ) (study aims 1 and 2)

Chronotype was calculated from the MCTQ, which assesses the time of sleep onset and sleep end on work days and free days over the preceding 2 weeks. A half-way point between sleep onset and sleep end (i.e. mid-sleep) was calculated for both work and free days, reflecting the subject’s chronotype. For the purposes of this study, the chronotype (MSFsc) was expressed as mid-sleep on free days, with correction for sleep deficit accumulated during work days (see reference [9] for detailed calculation).

##### Checklist Individual Strength (CIS)-20 (study aim 2)

Fatigue was assessed with the CIS-20 [10]. From this self-assessment questionnaire, we determined the total score (20 items), and the subscale score subjective fatigue (8 items). Higher scores reflect higher levels of fatigue.

##### RAND-36 (study aim 2)

Health-related quality of life (HR-QoL) was assessed with this self-assessment questionnaire (36 items). Nine subscale scores were determined. Transformed values ranged from 0 to 100, with higher values corresponding to better health-related quality of life.

##### Holland Sleep Disorder Questionnaire (HSDQ) (study aim 3)

This self-assessment questionnaire was used to explore the presence of sleep disorders based on the International Classification of Sleep Disorders-2 [11]. Six categories of sleep disorders are distinguished: circadian rhythm sleep disorders, insomnia, parasomnia, hypersomnia, sleep-related breathing disorders, and sleep-related movement disorders [12].

#### Norm group data for timing sleep-wake behaviour in general population (study aim 1)

Norm group data on chronotype were obtained from a large Dutch general population database, for which the MCTQ was completed online from 2003 to 2015; for more details, see the reference article [13]. Individuals from the general population (age range: 18–80 years) who filled in this questionnaire in the period between September 24 and October 24 (daylight saving time) were selected for the current study to reflect a similar data collection period as the RA patient sample. From this norm group, the average values of MSFsc were available for females and males separately, per 2 years, from 18 to 60 years (i.e. averages from 18 to 20 years, 20–22 years, etc.), 60 to 65 years, and 65 to 80 years.

**Fig. 1:**
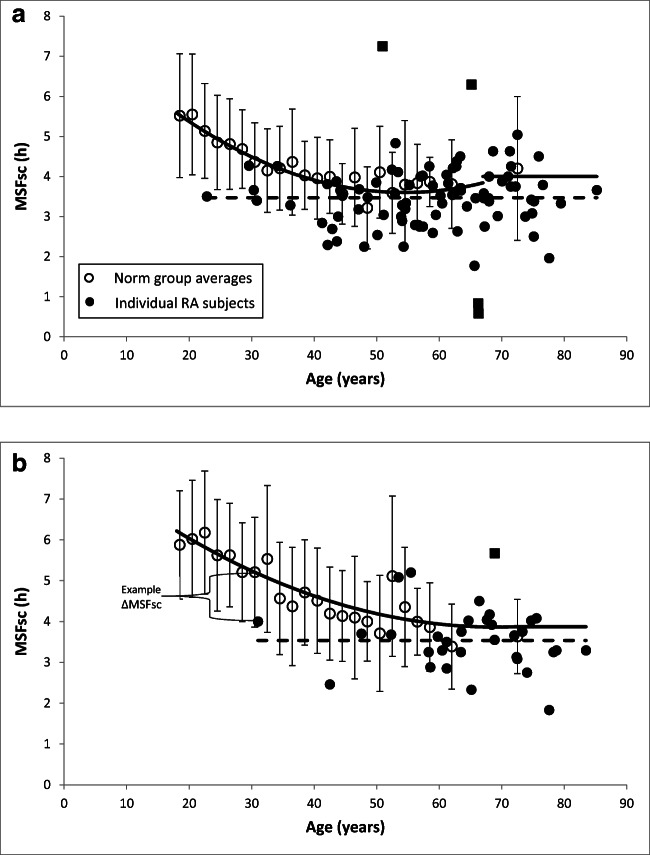
Plot of the MSFsc values in females (**a**) and males (**b**). Open circles represent the average values of the norm group subjects (the average values were available per 2 years from 18 to 60 years, and from 60 to 65 years and 65 to 80 years). Error bars show the standard deviations. The solid lines represent the MSFsc,norm values used (see text for formulas). The closed circles represent the MSFsc of each individual subject with RA; the squares are outliers. The dotted lines show the average MSF in our RA sample for females (i.e. 3:28 h:mm) and males (i.e. 3:32 h:mm). ΔMSFsc was determined by subtracting MSFsc,norm from MSFsc and expressing the number of minutes by which the MSFsc of a certain subject deviates from the MSFsc of the average subject with the same age and gender (see example in **b**)

Since chronotype is dependent on age and gender, we calculated age- and gender-related norm values (MSFsc,norm) by constructing a best fit line (polynomial trend line, order 2) for MSFsc as a function of age, separately for females (*n* = 665; *R*^2^ = 83%) and males (*n* = 1030; *R*^2^ = 90%) with this norm data (see the solid lines in Fig. 1). During evaluation of these best fit lines of the norm data, we noticed an unnatural increase in MSFsc at older ages, which has not been noted in the literature [14]. There was therefore a risk that we might find the hypothesised earlier sleep-wake behaviour in RA merely because of these unnaturally high values in the norm group at older ages. To minimise this risk, we fixed the MSFsc,norm values for older ages at a lower (earlier) value: at 4:00 h from the age of 67 years for females, and at 3:52 h from the age of 72 years for males (see Fig. 1 a and b). This resulted in the following formulas for MSFsc,norm:

Females

$$ {\displaystyle \begin{array}{c}\mathrm{Subjects}\ \mathrm{age}\le 67\ \mathrm{y}:\mathrm{MSFsc},\operatorname{norm}\ \left(\mathrm{in}\ \mathrm{hours}\right)=0.0015\times {\mathrm{age}}^2\hbox{--} 0.1631\times \mathrm{age}+8.0348\\ {}\ \mathrm{Subjects}\ \mathrm{age}>67\ \mathrm{y}:\mathrm{MSFsc},\operatorname{norm}\ \left(\mathrm{in}\ \mathrm{hours}\right)=4.00\end{array}} $$

Males

$$ {\displaystyle \begin{array}{c}\mathrm{Subjects}\ \mathrm{age}\le 72\ \mathrm{y}:\mathrm{MSFsc},\operatorname{norm}\ \left(\mathrm{in}\ \mathrm{hours}\right)=0.0009\times {\mathrm{age}}^2\hbox{--} 0.1244\times \mathrm{age}+8.1618\\ {}\mathrm{Subjects}\ \mathrm{age}>72\ \mathrm{y}:\mathrm{MSFsc},\operatorname{norm}\ \left(\mathrm{in}\ \mathrm{hours}\right)=3.87\ \left(=3:52\ \mathrm{h}:\mathrm{mm}\right)\end{array}} $$

#### Data from medical records (study aim 2)

Disease activity data were extracted from medical records of the most recent EAC measurement preceding the completion of the questionnaires (on average 5.3 months [median 5.6 months] ± SD 2.8 months [range: between 7 days and 10 months]). Disease activity was assessed using the Disease Activity Score (DAS) based on a 44-joint score for swollen joint count, and a Ritchie Articular Index (53 joints) for joint pain on palpation.

From the same EAC measurement, the duration of morning stiffness was assessed as an ordinal variable with seven categories: no morning stiffness, 1–30 min, 30–60 min, 1–2 h, 2–4 h, > 4 h, whole day. The presence of morning stiffness was examined in the current study as a categorical variable (yes/no) in two ways: (1) no morning stiffness vs. the other categories; (2) < 1 h vs. > 1 h.

### Calculations and statistics

Data were analysed with SPSS Statistics for Windows (Version 23, IBM Corporation, Armonk, NY, USA).

#### Study aim 1

For each subject in our RA sample, we calculated the difference between (1) the MSFsc based on the MCTQ filled in by the subject, and (2) the MSFsc,norm based on the age and gender of the subject, using the formulas presented above. This difference, ΔMSFsc, expresses the number of minutes by which the MSFsc of a certain RA subject deviates from the MSFsc of the norm group. A ΔMSFsc of zero means no difference; a positive value indicates a later chronotype; and a negative value indicates an earlier chronotype for the subject with RA in comparison with the norm group.

##### Main analysis

A one-sample *t* test was conducted to examine whether ΔMSFsc deviated significantly from 0. A *p* value < 0.05 was considered significant. Cohen’s *d* was calculated as a measure of effect size.

##### Additional exploratory analyses

As the norm data were dominated by subjects of a younger age, we performed two additional exploratory analyses including only the youngest part of our sample: (1) one analysis excluded females > 67 years and males > 72 years (i.e. the ages at which the norm values were fixed); and (2) one analysis excluded subjects whose age lays above the mean age of the RA sample (i.e. 60 years).

#### Study aim 2

##### Main analyses

To relate chronotype to disease outcomes, we created four equally sampled groups based on MSFsc (from early to late chronotype): ‘≤ 3:00 h’ (*n* = 29); ‘3:00–3:30 h’ (*n* = 30); ‘3:31–4:00 h’ (*n* = 34); and ‘>4:00 h’ (*n* = 28). MSFsc was categorised for this analysis to keep open the possibility of a non-linear relationship.

To compare the chronotype groups on disease outcome measures, we performed one-way ANOVA analyses (CIS-scores), Kruskal-Wallis analyses (HR-QoL subscale scores and DAS scores), and chi-square analyses (presence of MS). As these analyses were explorative, an uncorrected *p* value of < 0.05 was considered significant.

##### Additional exploratory analyses

For additional exploratory purposes, the relations between the *continuous* measure of chronotype (i.e. MSFsc) and the outcomes were also explored.

In addition, we explored the relation between ∆MSFsc and outcomes. ∆MSFsc was considered both a continuous and a categorical variable (compared to the norm: ≥ 1 h earlier (*n* = 25); 1 h to 15 min earlier (*n* = 44); 15 min earlier to 15 min later (*n* = 30); and > 15 min later (*n* = 22).

## Results

### Subjects

Of the patients invited by a postal letter (*n* = 178), 34% participated (27% on paper, 7% online). Of the patients invited by email (*n* = 127), 54% participated (53% online, 1% on paper [after request]). This resulted in 129 subjects (response rate: 42%; paper: *n* = 49, online: *n* = 80). Eight subjects were excluded because the MSFsc could not be determined due to incomplete MCTQ (*n* = 3), or because they had engaged in shift work in the 3 months before the measurement (*n* = 5).

The mean age of the remaining 121 subjects was 60 years (SD: 12, range: 23–85 years), with 88 females and 33 males. The participants had a median disease duration of 6 years (IQR: 10), and the median Disease Activity Score (DAS44) was 1.6 (IQR: 1.2). Fifty-three percent of the sample worked; the median weekly number of work days of those subjects was four (IQR: 2). Sixteen percent of the subjects used an alarm clock on free days. The median time spent outdoors was 2.0 h/day (IQR: 1.8). Corticosteroids were used by eight participants. For more patient characteristics, see Online Resource 1.

### Study aim 1

#### Descriptives

The MSFsc and ΔMSFsc of five subjects were marked as unusually extreme outliers (3 extremely late and 2 extremely early); these subjects were therefore excluded for the one-sample *t* test and the subsequent descriptives. The MSFsc and ΔMSFsc were normally distributed with an MSFsc mean ± SD of 3:29 h:mm ± 41 min. See Fig. 1 for the subjects’ individual MSFsc values. Table 1 gives an overview of the main sleep variables as obtained with the MCTQ.

**Table 1:** Main sleep variables as obtained with the MCTQ (*n* = 116)

Variable		Mean (h:mm); SD (min)
RA subjects		
Sleep onset	Free days	23:37; 50
	Week average	23:30; 48
Sleep end	Free days	7:36; 68
	Week average	7:16; 58
Sleep duration	Free days	7:59; 76
	Week average	7:46; 71
MSFsc		3:29; 41
RA subjects as compared with norm group		
ΔMSFsc		− 0:23; 42

#### Background analyses

MSFsc was strongly related to ΔMSFsc (*r*_s_ = .916, *n* = 116, *p* < 0.001). An earlier MSFsc was related to an earlier time point of sleep onset on free days (*r*_s_ (114) = .686, *p* < 0.001), an earlier time point of sleep end on free days (*r*_s_ (114) = .755, *p* < 0.001), and a shorter sleep duration on free days (*r*_s_ (114) = .227, *p* = 0.014). Online Resource 2 shows all correlations between the main sleep variables.

The sleep variables presented in Table 1 are not related to age, gender, or disease duration (*p* > 0.05), except for the sleep end (averaged over the week), which was positively related to age (*r*_s_ (114) = .203, *p* = 0.029).

#### Main analysis

A one-sample *t* test showed that the ΔMSFsc deviated significantly from 0 (*t*(115) = − 5.901, *p* < 0.001, *d* = 0.55); the MSFsc was on average 23 min (95% CI, 15 to 31 min) earlier in RA patients than in the norm group.

#### Additional exploratory analyses

The two analyses including only the youngest part of our sample (see “Materials and methods”) confirmed the findings of the main analysis, showing an earlier MSFsc in the RA patients than in the norm group: on average (1) 21 (95% CI, 12 to 30), and (2) 26 (95% CI, 14 to 38) min (*p* < 0.001).

Another exploratory analysis showed that, without exclusion of the five subjects with extreme early or late chronotypes, also an average significant deviation was found (of 21 min [95% CI, 11 to 31, *p* < 0.001]).

### Study aim 2

#### Main analysis

Table 2 shows the descriptives and statistics of disease activity, reported morning stiffness, fatigue, and HR-QoL for each chronotype group. None of these measures differed to a statistically significant extent between the four chronotype groups (*p* > 0.05; see Table 1).

**Table 2:** Descriptives of disease activity, morning stiffness, fatigue, and health-related quality of life and test statistics of the comparison of the four chronotype groups (*n* = 121)

	All	Group					
Outcome measure		< 3.0 h	3.0–3.5 h	3.5–4.0 h	> 4.0 h	Test statistic	*p*
	Median (IQR)	Mean rank				*χ*^2^(3)	
Disease activity (DAS44)	1.62 (1.17)	60	61	63	59	0.264	0.967
Visual analogue scale (0–100 mm) pain, mm	20 (50)	68	64	52	62	3.737	0.291
Visual analogue scale (0–100 mm) general well-being, mm	30 (30)	66	58	59	62	0.987	0.804
Erythrocyte sedimentation rate, mm/h	9 (16)	58	65	59	62	0.917	0.821
Ritchie tender joint index	1 (3)	60	58	67	58	1.663	0.645
Number of swollen joints	0 (1)	53	56	70	65	5.729	0.126
Reported morning stiffness^a^	*n* present (%)	*n* present (%)				*χ*^2^(3)	
Morning stiffness presence^b^	65 (60%)	16 (59%)	18 (67%)	15 (48%)	16 (67%)	2.678	0.444
Morning stiffness > 1 h	12 (11%)	3 (11%)	3 (11%)	3 (10%)	3 (12%)	0.111	0.990
Fatigue (Checklist Individual Strength)	Mean ± SD	Mean ± SD				*F*(3,117)	
Checklist Individual Strength-20	72 ± 26	75 ± 21	69 ± 22	77 ± 28	68 ± 31	0.958	0.415
Checklist Individual Strength-8	34 ± 12	36 ± 12	31 ± 11	35 ± 13	32 ± 14	1.116	0.326
Health-related quality of life (RAND-36)^c^	Median (IQR)	Mean rank				*χ*^2^(3)	
Subscale general health perception	50 (30)	56	64	53	68	3.543	0.315
Subscale health change	50 (25)	66	57	56	64	1.997	0.573
Subscale role functioning; physical problems	50 (100)	61	59	56	64	1.009	0.799
Subscale role functioning; emotional problems	100 (50)	61	64	58	61	0.669	0.880
Subscale energy/fatigue	55 (30)	61	66	55	61	1.586	0.663
Subscale pain	67 (35)	59	53	60	62	0.273	0.965
Subscale physical functioning	75 (34)	59	61	64	59	0.442	0.931
Subscale social functioning	75 (25)	52	65	56	69	4.459	0.216
Subscale mental health	80 (24)	58	62	53	71	4.689	0.196

^a^12 subjects missing; ^b^1–30 min, 30–60 min, 1–2 h, 2–4 h, > 4 h, whole day vs. no morning stiffness; ^c^0–2 subjects missing

#### Additional exploratory analyses

No relationship was found between the continuous measure of chronotype and the outcomes (*p* > 0.05).

The analyses exploring the relation between ∆MSFsc and outcomes showed an indication for a relationship between the number of swollen joints and ∆MSFsc (categorical ∆MSFsc: *X*^2^(3) = 12.451; *p* = 0.006; continuous ∆MSFsc: *r*_s_ = .224, *p* = 0.016). The relation was unexpectedly in favour of subjects with a much earlier chronotype as compared to subjects with MSFsc values close to those in the norm group (see Online Resource 3).

### Study aim 3

According to the Holland Sleep Disorder Questionnaire, 37% of the subjects presented one or more sleep disorders; a circadian rhythm sleep disorder was present in 4% of the subjects (see also Online Resource 4).

## Discussion

A previous study showed an earlier timing of the circadian rhythm, reflected by earlier peaks in serum melatonin levels during the night [4]. In line with this, in the current study, we found (as expected) an earlier chronotype in patients with RA than in subjects from the general population.

An earlier chronotype, reflected in the current study by mid-sleep, could result from earlier sleep onset and/or earlier sleep end. Although deviations in sleep onset and sleep end were not explored in the current study, patients’ accounts suggested that they both fall asleep and awaken earlier than desired.

Although there are indications that patients with RA have both an earlier chronotype (this study) and an earlier internal circadian pacemaker (indicated by earlier peaks of melatonin during the night [4]), it is unclear how those two rhythms are related within an individual patient. It would be interesting to examine the time between melatonin onset and bedtime/sleep onset time (i.e. phase angle) to find out whether patients with RA go to bed and/or start sleeping at the optimal time after their melatonin levels start rising [15]. A possible misalignment might be related to patient-reported outcomes (e.g. fatigue) and disease activity.

In the main analyses, no relations were found between chronotype and disease activity, reported morning stiffness, fatigue, or HR-QoL. One additional explorative analysis gave us an indication for a possible relation: subjects with a much earlier chronotype than the norm group had unexpectedly on average less number of swollen joints than those with a chronotype close to the subjects in the norm group. As this was an additional explorative analysis and uncorrected *p* values were used, this finding should be confirmed in another study.

Possible explanations for the absence of relations between chronotype and disease activity include (1) the subjective nature of some of the measurements used (e.g. fatigue, HR-QoL, and sleep times), which reduces the comparability between subjects; (2) the fact that measurements of chronotype and disease activity were not performed on the same day (on average there were 5 months between these measurements, which complicates the interpretation of a correlation analysis); (3) the presence of several fatigue subtypes; (4) the presence of pain; (5) the low average disease activity in our sample, which reduces the variability in this measure; and/or (6) the possible influence of pharmacological medication (e.g. corticosteroids) on both disease activity and circadian rhythmicity (note: corticosteroids were used by very few patients in our sample).

Alternatively, it is possible that the outcomes are related not to chronotype but to the timing of the internal circadian pacemaker. Although chronotype is influenced by the internal circadian pacemaker, several other factors are also involved: social factors (e.g. work and social activities); environmental factors (e.g. time point and amount of light exposure and physical activity); and the rate of the accumulation (falling asleep) and dissipation (awaking) of the homeostatic sleep pressure.

Therefore, for future studies, it might be fruitful to examine whether measurements of the circadian rhythm (i.e. endogenous measures such as the timing of the internal circadian pacemaker as measured with dim light melatonin onset) are related to disease activity and patient-reported outcomes. Furthermore, it would be interesting to investigate the earlier timing of the internal circadian pacemaker and chronotype in relation to more specific patient-reported measures (rather than the general measure of fatigue applied in the current study) such as the feeling of sleepiness in the early evening and sleeplessness in the early morning which could influence HR-QoL.

Given that a correlational study does not establish causality, we are unable to draw conclusions about whether an earlier chronotype has *an effect* on disease activity and patient-related outcomes. It would be of interest to examine whether experimentally delaying the circadian rhythm in patients with an earlier rhythm—thus establishing a later rhythm that is possibly more aligned with the external light-dark cycle—would make the patients feel less fatigued (possibly due to better sleep quality and/or a reduction of inflammatory activity during the night). One way to induce a delay of this kind would be through light exposure in the evening [16]. Rao et al. [17] elaborate extensively on the promising role of circadian realignment in improving RA outcomes.

In our sample, the proportion of sleep disorders among the RA participants was comparable to that of a general Dutch population [12]. Our finding of an earlier chronotype could indicate the presence of an advanced sleep-wake phase as defined by the ICSD. However, the questionnaire used for measuring circadian rhythm sleep disorders does not explicitly measure the *advanced* sleep-wake phase disorder. This could explain the fact that a circadian rhythm sleep disorder was found in only a small number of the participants, which was comparable to the general Dutch population [12]. Furthermore, patients with RA may not have a circadian rhythm sleep *disorder*, but only an advanced chronotype (without this being a disorder).

The subjects in the RA sample had a mean age of 60 years; however, the norm group included only fifty individuals above this age. Therefore, the norm values used in the age group above 60 years may be less reliable. This uncertainty is increased by the fact that our best fit lines show an unnatural increase in chronotype (i.e. later) at older ages, whereas earlier studies show that people’s chronotype increases from approximately 20 years [14]. To avoid the risk of finding the hypothesised earlier chronotype in RA just because of the unnaturally high values at older ages in the norm group, we fixed the values of the norm group at older ages at a lower (earlier) value, and still found an earlier chronotype. This finding was confirmed by exploratory analyses including only subjects of a lower age. Nevertheless, it is recommended for future studies to use a norm group with more persons in the age of interest (e.g. partners of patients).

Only 42% of the patients invited to participate decided to do so; this may have led to selection bias. As patients were informed in advance what the study was about, it is possible that patients with sleep concerns were more willing to participate. This could have affected the generalisability of the results.

In summary, we found an earlier chronotype in RA; this is in line with a previous finding that the timing of the internal circadian pacemaker is earlier in RA patients than in people from the general population. In this correlational study, an earlier chronotype does not seem to be related to several debilitating symptoms observed in RA, such as fatigue. An experimental study is needed to examine whether delaying the circadian rhythm has a positive influence on fatigue and other outcomes. Furthermore, future studies should examine whether those symptoms are related to measurements of circadian timing (e.g. dim light melatonin onset). This insight can improve our understanding of the pathophysiology of RA and contribute to the exploration of new treatment possibilities.

## Supplementary information

ESM 1 (DOCX 42 kb)

## Data Availability

The datasets generated and/or analysed during the current study will be made available in DataverseNL (dataverse.nl) after publication. They are also available from the corresponding author on reasonable request.
