# Correction to: Earlier chronotype in patients with rheumatoid arthritis

**DOI:** 10.1007/s10067-021-05606-w

**Published:** 2021-01-28

**Authors:** G. Esther A. Habers, Annette H. M. van der Helm-van Mil, Dieuwke S. Veldhuijzen, Cornelia F. Allaart, Erno Vreugdenhil, Daniëlle E. J. Starreveld, Tom W. J. Huizinga, Andrea W. M. Evers

**Affiliations:** 1 Health, Medical, and Neuropsychology Unit, Institute of Psychology, Leiden University, Wassenaarseweg 52, 2333 AK Leiden, the Netherlands; 2 Leiden Institute for Brain and Cognition, Leiden, the Netherlands; 3 Department of Rheumatology, Leiden University Medical Center, Leiden, the Netherlands; 4 Department of Cell and Chemical Biology/Department of Dermatology, Leiden University Medical Center, Leiden, the Netherlands; 5 Department of Psychosocial Research and Epidemiology, Netherlands Cancer Institute, Amsterdam, the Netherlands; 6 Department of Psychiatry, Leiden University Medical Center, Leiden, the Netherlands; 7 Industrial Design Engineering, Delft University of Technology, Delft, the Netherlands; 8 Erasmus School of Health Policy and Management, Erasmus University, Rotterdam, the Netherlands

**Correction to: Clinical Rheumatology**

10.1007/s10067-020-05546-x

The original version of this article contained error.

In the last part of the formula under section “Norm group data for timing sleep-wake behaviour in general population (study aim 1)” was described **“(=33:52 h:mm)**”, it should have been **“ (= 3:52 h : mm)”**. The original article has been corrected.

