# Supplementary material for: Earlier chronotype in patients with rheumatoid arthritis
Source: Clin Rheumatol. 2021 Jan 16;40(6):2185–92. doi: 10.1007/s10067-020-05546-x (PMC8121723; doi:10.1007/s10067-020-05546-x)
Supplement: Supplementary file 1 — (DOCX 42 kb) [file 10067_2020_5546_MOESM1_ESM.docx]

**Online Resources**

**Journal:** Clinical Rheumatology

**Author names**: G. Esther A. Habers, Annette H.M. van der Helm-van Mil, Dieuwke S. Veldhuijzen, Cornelia F. Allaart, Erno Vreugdenhil, Daniëlle E.J. Starreveld, Tom W.J. Huizinga, Andrea W.M. Evers.

**Title:** Earlier chronotype in patients with rheumatoid arthritis.

**Corresponding author:**G.E.A. Habers
Health, Medical, and Neuropsychology Unit, Institute of Psychology, Leiden University
Wassenaarseweg 52
2333 AK Leiden
The Netherlands
T: +31 (0)71 527 5236
F: +31 (0)71 527 3619
E: g.e.a.habers@fsw.leidenuniv.nl

**Online Resource 1.**

***Table Online Resource 1.*** Patient characteristics (n=121).

| **Characteristic** | **Total**  **(*n*=121)** | **Female (*n*=88)** | **Male**  **(*n*=33)** |
| --- | --- | --- | --- |
| **Age years, *mean (SD)*** | 60.2 (12.5) | 58.5 (12.6) | 64.8 (11.1) |
| **Disease duration years, *median (IQR)*** | 6.4 (10.4) | 7.4 (11.2) | 5.7 (7.4) |
| **Marital status, *n (%) ^a^*** | - | - | - |
| Single | 15 (13) | 13 (15) | 2 (6) |
| Together with partner, not living together | 3 (3) | 2 (2) | 1 (3) |
| Living together with partner | 7 (6) | 5 (6) | 2 (6) |
| Married or registered partners | 89 (72) | 64 (73) | 25 (78) |
| Divorced or separated | 5 (4) | 4 (5) | 1 (3) |
| Widow(er) | 1 (1) | 0 (0) | 1 (3) |
| **Education, *n (%) ^b^*** | - | - | - |
| No | 1 (1) | 0 (0) | 1 (3) |
| Primary school | 7 (6) | 4 (5) | 3 (9) |
| Preparatory middle-level applied education ^c^ | 46 (38) | 36 (41) | 10 (30) |
| Middle-level applied education | 19 (16) | 14 (16) | 5 (15) |
| Higher general continued education/ preparatory scholarly education | 8 (7) | 5 (6) | 3 (9) |
| Higher professional education | 24 (20) | 16 (18) | 8 (24) |
| Scientific education | 15 (12) | 12 (14) | 3 (9) |
| **Work/study, *n (more answers possible)*** | - | - | - |
| Unemployed | 2 | 1 | 1 |
| Paid work | 51 | 42 | 9 |
| Unpaided work | 6 | 5 | 1 |
| School or study | 3 | 2 | 1 |
| Housekeeping | 25 | 25 | 0 |
| Sickness Benefit Act/Work incapacitated | 20 | 16 | 4 |
| Retired | 46 | 25 | 21 |
| **Number of workdays***, n (%)* |  |  |  |
| No workdays | 57 (47%) | 38 (43%) | 19 (58%) |
| 1-2 workdays | 9 (7%) | 6 (7%) | 3 (9%) |
| 3-5 workdays | 51 (42%) | 42 (48%) | 9 (27%) |
| 6-7 workdays | 4 (3%) | 2 (2%) | 2 (6%) |
| **Alarm clock on free days, yes***, n (%)* | 19 (16%) | 14 (16%) | 5 (15%) |
| **Hours spend outdoors a day***, median (IQR)* |  |  |  |
| Free days ^d^ | 2.1 (2.0) | 2.0 (2.0) | 2.5 (2.0) |
| Work days | 1.4 (1.5) | 1.4 (1.5) | 1.4 (2.3) |
| Average work and free days ^d^ | 2.0 (1.8) | 1.9 (1.9) | 2.0 (3.0) |

^a^ Missing: 1; ^b^ Missing: 1; ^c^ Vocational education and Secondary education taken together; ^d^ Data from five females missing.

**Online Resource 2.**

***Table Online Resource 2.*** Correlations between main sleep variables.

|  |  |  | **Sleep onset** |  | **Sleep end** |  | **Sleep duration** |  | **MSFsc** | **ΔMSFsc** |
| --- | --- | --- | --- | --- | --- | --- | --- | --- | --- | --- |
|  |  |  | *Free days* | *Week average* | *Free days* | *Week average* | *Free days* | *Week average* |  |  |
| **Sleep onset** | *Free days* |  | X | **.952***** | **.212*** | NS | **-.407***** | **-.496***** | **.686******* | **.667***** |
|  | *Week average* |  |  | X | NS | **.200 *** | **-.423***** | **-.489***** | **.649***** | **.624***** |
| **Sleep end** | *Free days* |  |  |  | X | **.764***** | **.738***** | **.579***** | **.755***** | **.674***** |
|  | *Week average* |  |  |  |  | X | **.587***** | **.678***** | **.748***** | **.655***** |
| **Sleep duration** | *Free days* |  |  |  |  |  | X | **.882***** | **.227*** | NS |
|  | *Week average* |  |  |  |  |  |  | X | NS | NS |
| **MSFsc** |  |  |  |  |  |  |  |  | X | **.916***** |

Spearman’s rho correlation coefficient. **p* < .05, ***p* < .01, ****p* < .001, NS: not significant (*p* > .05)

**Online Resource 3.**

***Table Online Resource 3.*** The relation between chronotype (i.e. MSFsc) and chronotype deviation (∆MSFsc) and number of swollen joints (*n*=121).

| **Chronotype measure** | **Groups** | **n** | **Mean rank** | **Test statistic** | **p** |
| --- | --- | --- | --- | --- | --- |
| ***Main analysis*** |  |  |  |  |  |
| *MSFsc, categorical* | <3.0h | 29 | 53 | *χ^2^(3) =* 5.729 | .126 |
|  | 3.0-3.5h | 30 | 56 |  |  |
|  | 3.5-4.0h | 34 | 70 |  |  |
|  | >4.0h | 28 | 65 |  |  |
|  |  |  |  |  |  |
| ***Additional exploratory analyses*** |  |  |  |  |  |
| *MSFsc, continue* | NA | 116 |  | *r*_s_ = .145 | .121 |
|  |  |  |  |  |  |
| *∆MSFsc, categorical* | ≥1 h earlier | 25 | 47 ^a,b^ | *χ^2^(3) =* 12.451 | **.006** |
|  | 1 h to 15 minutes earlier | 44 | 56 ^c^ |  |  |
|  | 15 minutes earlier to 15 minutes later | 30 | 74 ^a,c^ |  |  |
|  | >15 minutes later | 22 | 69 ^b^ |  |  |
|  |  |  |  |  |  |
| *∆MSFsc, continue* | NA | 116 |  | *r*_s_ = .224 | **.016** |

^a^ Pairwise comparisons: uncorrected *p*-value: .002
^b^ Pairwise comparisons: uncorrected *p*-value: .014
^c^ Pairwise comparisons: uncorrected *p*-value: .021
Other pairwise comparisons had an uncorrected *p*-value > .05

Median (IQR) of number of swollen joints is 0 (1).

*r*_s_: Spearman’s rho correlation coefficient.


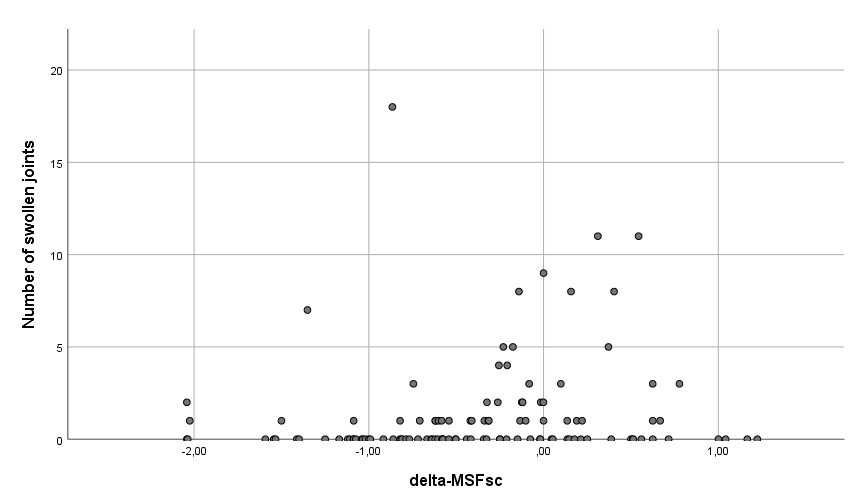


***Figure Online Resource 3.*** Relation between ∆MSFsc and number of swollen joints.

**Online Resource 4.**

***Table Online Resource 4.*** Presence of sleep disorders determined with the Holland Sleep Disorder Questionnaire.

| **Sleep disorder** | **Total**  **(*n*=124)** | **Female (*n*=91)** | **Male**  **(*n*=33)** |
| --- | --- | --- | --- |
| **One or more sleep disorders, *n (%)*** | 46 (37%) | 36 (40%) | 10 (30%) |
| Sleep related movement disorder | 20 (16%) | 15 (16%) | 5 (15%) |
| Sleep related breathing disorder | 14 (11%) | 10 (11%) | 4 (12%) |
| Hypersomnia | 9 (7%) | 6 (7%) | 3 (9%) |
| Insomnia | 7 (6%) | 6 (7%) | 1 (3%) |
| Parasomnia | 3 (2%) | 1 (1%) | 2 (6%) |
| **Circadian rhythm sleep disorder** | **5 (4%)** | **4 (4%)** | **1 (3%)** |

Note. Five missings from original sample size.
